# Supplementary figures and images for: Lipoarabinomannan from Mycobacterium indicus pranii shows immunostimulatory activity and induces autophagy in macrophages
Source: PLoS One. 2019 Oct 24;14(10):e0224239. doi: 10.1371/journal.pone.0224239 (PMC6812838; doi:10.1371/journal.pone.0224239)

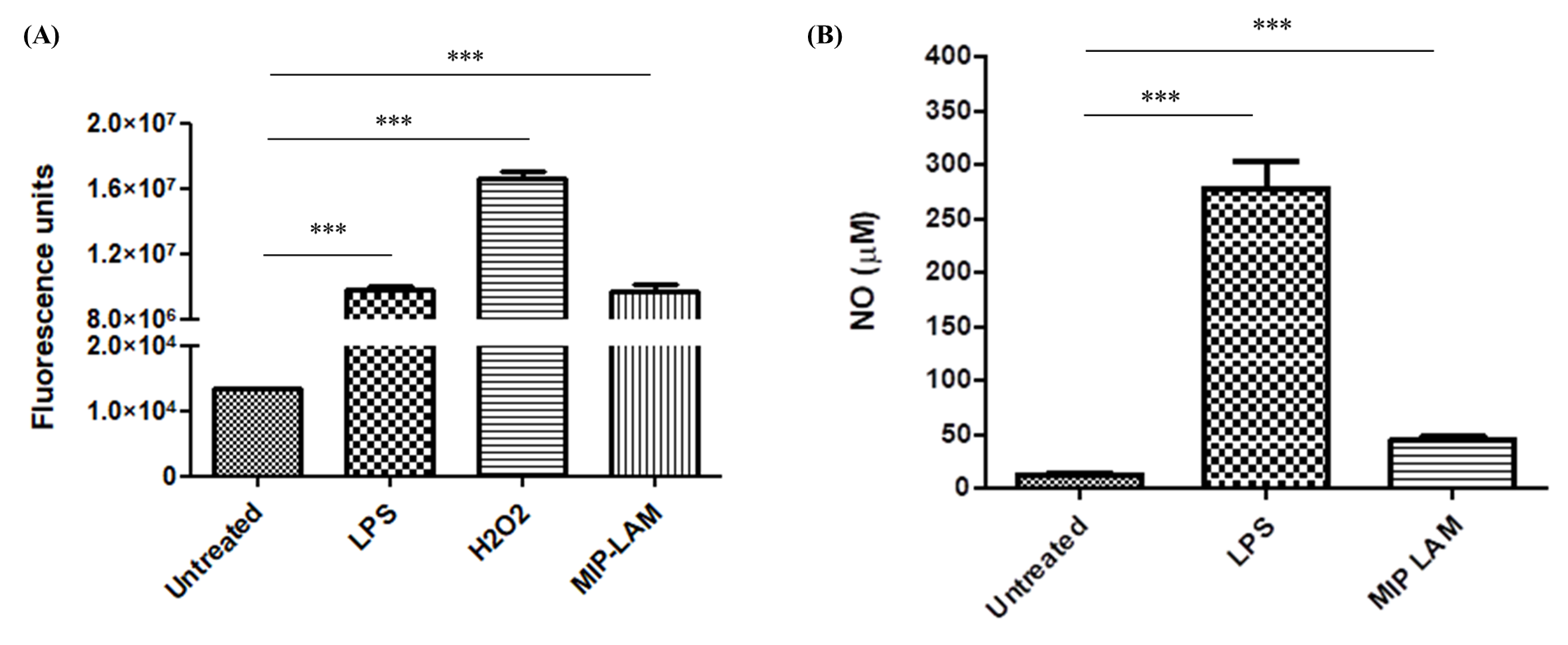

Supplement: S1 Fig — (A) RAW macrophages were left unstimulated or stimulated with LPS (1 μg/mL) or MIP-LAM (5 μg/mL) for 24 h. Supernatant was collected and estimated for NO with Griess reagent. (B) ROS in macrophages was determined by using 2',7'-dichlorodihydrofluorescein diacetate (H2DCFDA) dye. Fluorescence was determined using fluorimeter excitation at 490 nm and emission at 524 nm. H2O2 (100 μM) was used as a positive control. ***: P<0.0001. (TIF) [file pone.0224239.s001.tif]

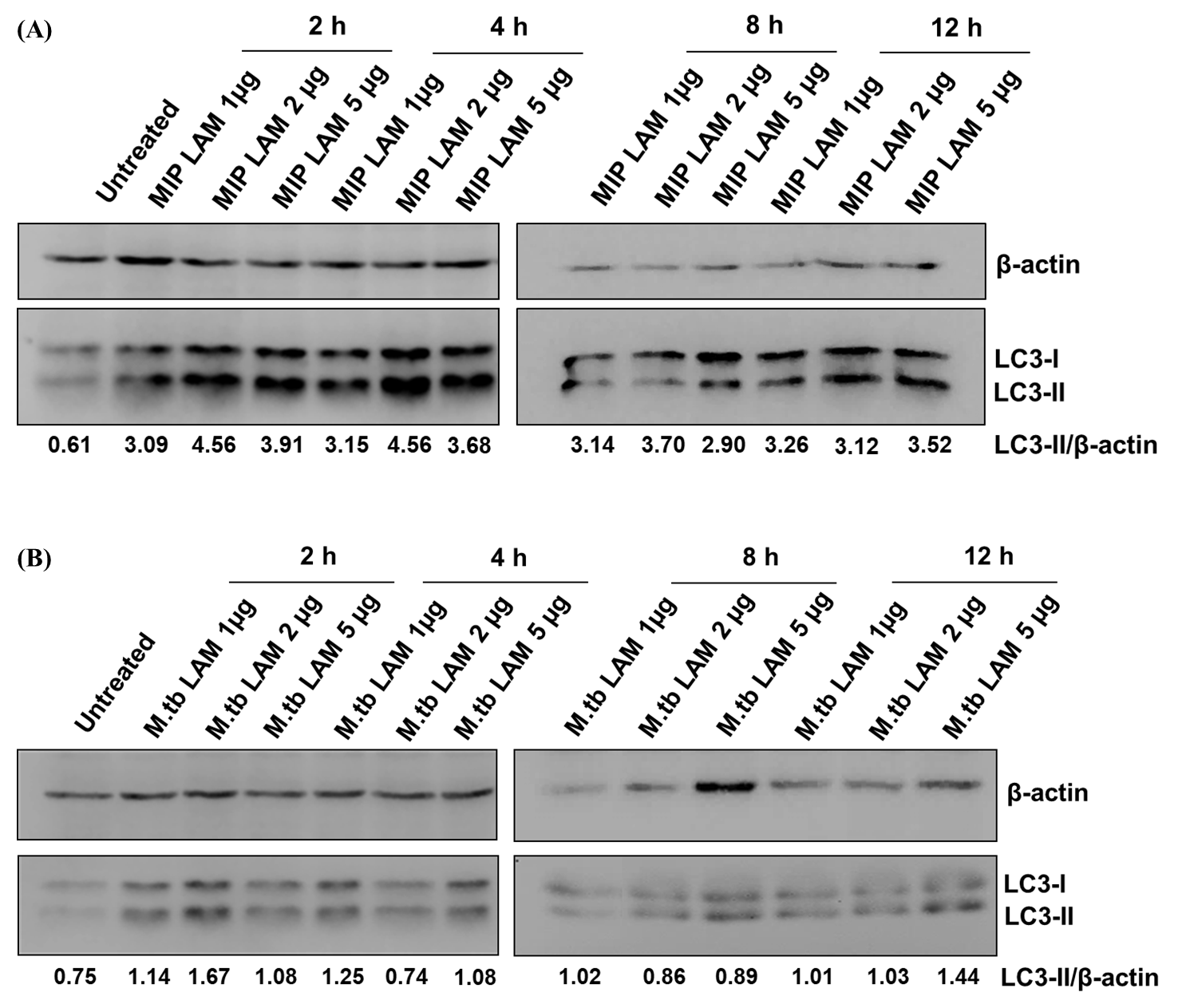

Supplement: S2 Fig — RAW 264.7 macrophages were stimulated with various concentrations of MIP-LAM / M.tb-LAM for 2, 4, 8 and 12 h. Shown are the Western blots for the indicated time points. (TIF) [file pone.0224239.s002.tif]
