# Supplementary material for: Lipoarabinomannan from Mycobacterium indicus pranii shows immunostimulatory activity and induces autophagy in macrophages
Source: PLoS One. 2019 Oct 24;14(10):e0224239. doi: 10.1371/journal.pone.0224239 (PMC6812838; doi:10.1371/journal.pone.0224239)
Supplement: Attachment — Submitted filename: Response to reviewers.docx [file pone.0224239.s004.docx]

**Rebuttal**

Additional experiments performed for the revision are important and should be included, described and analyzed in the revised manuscript.

**Response:** As per the suggestions, we have included the additional experiments and their results in the revised manuscript.
